# Advancement in Biosensor Technologies of 2D MaterialIntegrated with Cellulose—Physical Properties

**DOI:** 10.3390/mi15010082

**Published:** 2023-12-30

**Authors:** Ghazaleh Ramezani, Ion Stiharu, Theo G. M. van de Ven, Vahe Nerguizian

**Affiliations:** 1 Department of Mechanical, Industrial, and Aerospace Engineering, Concordia University, Montreal, QC H3G 1M8, Canada; ghazaleh.ramezani@mail.concordia.ca; 2 Department of Chemistry, McGill University, 801 Sherbrooke St. West, Montreal, QC H3A 0B8, Canada; theo.vandeven@mcgill.ca; 3 Department of Electrical Engineering, École de Technologie Supérieure, 1100 Notre Dame West, Montreal, QC H3C 1K3, Canada; vahe.nerguizian@etsmtl.ca

**Keywords:** biosensor technologies, two-dimensional (2D) materials, nanocellulose, sensitivity improvement

## Abstract

This review paper provides an in-depth analysis of recent advancements in integrating two-dimensional (2D) materials with cellulose to enhance biosensing technology. The incorporation of 2D materials such as graphene and transition metal dichalcogenides, along with nanocellulose, improves the sensitivity, stability, and flexibility of biosensors. Practical applications of these advanced biosensors are explored in fields like medical diagnostics and environmental monitoring. This innovative approach is driving research opportunities and expanding the possibilities for diverse applications in this rapidly evolving field.

## 1. Introduction

This review paper discusses the recent advancements in biosensor technologies, focusing on how integrating two-dimensional (2D) materials with cellulose improves biosensing functionality. The primary focus is on applications in medical diagnostics and environmental monitoring. By exploring the unique properties of 2D materials like graphene and transition metal dichalcogenides, as well as their synergistic effects when combined with nanocellulose, this review highlights the practical applications of these advanced biosensors. It also addresses gaps in existing literature and suggests directions for future research to expand possibilities in this field.

### 1.1. Background: The Emergence of 2D Materials in Biosensor Technologies

The use of two-dimensional (2D) materials in biosensor technologies has revolutionized the field. Materials like graphene, transition metal dichalcogenides (MoS_2_ and WS_2_), hexagonal boron nitride (h-BN), and black phosphorus have a nanoscale thickness and distinct physical properties that might greatly enhance biosensor performance [1].

Graphene, with its exceptional electrical conductivity and mechanical strength, is widely recognized for its versatility in biosensors. Its planar structure and high electron mobility improve sensitivity and specificity, making it an ideal component [2].

Transition metal dichalcogenides, such as MoS_2_ and WS_2_, possess unique semiconductor properties due to their layered structure. These materials can interact with light and electrical fields, making them particularly suitable for biosensor applications that require precise electrical characteristics [3]. Also, studies enhance the role of 2D materials in cancer biosensors: a MoS_2_/Cu_2_O sensor for lung cancer detection [4], PEC biosensors for esophageal cancer [5], and a lab-on-chip design for broad cancer cell detection [6]. These highlight advancements in material use and detection techniques, aiding early cancer diagnosis.

**Figure 1:**
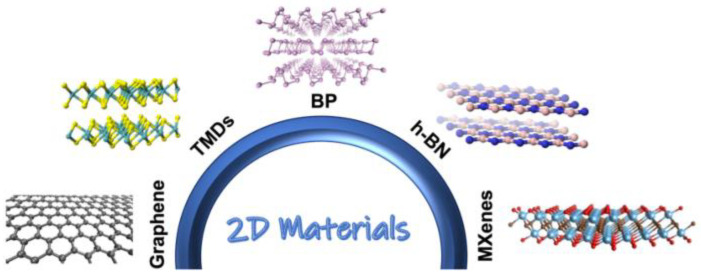
Major classes of 2D materials considered in this review: Graphene, TMDs, BP, h-BN, and MXenes. Reproduced with permission from [7].

Emerging as other 2D materials with unique optical and electronic properties, black phosphorus and hexagonal boron nitride (h-BN) offer new possibilities for expanding the range of biosensor functionalities [4]. In Figure 1, we review well-known 2D material-based sensors such as graphene, TMDs, h-BN, MXenes, and BP [7].

In addition, we review the synthesis and alteration procedures for these materials, which are crucial for customizing their characteristics to suit specific biosensor applications. These techniques include chemical vapor deposition, exfoliation, and functionalization. This knowledge provides a basis for comprehending the significant influence these materials have had on advancements in biosensor technology. Also, nanocellulose, derived from cellulose, has emerged as a promising material in biosensor technology and is discussed in the next section.

### 1.2. Overview of Nanocellulose

Nanocellulose, derived from cellulose, is a promising material in biosensor technology due to its biocompatibility and large surface area [8]. This section offers a comprehensive overview of different forms of nanocellulose, including cellulose nanocrystals, cellulose nanofibers, and bacterial cellulose. Furthermore, it explores the diverse applications of nanocellulose in the development of biosensors.

**Figure 2:**
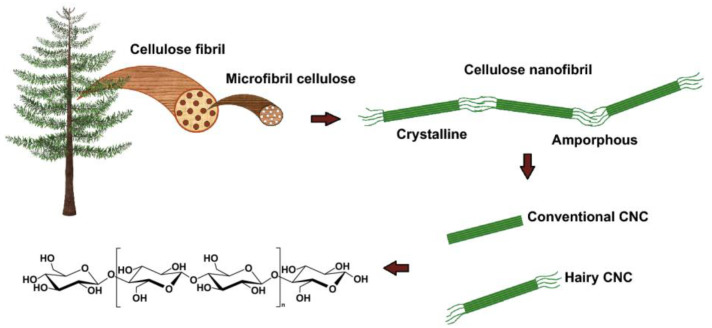
Schematic representation of cellulose structures from resources to molecular level. Reproduced with permission from [12].

- Cellulose nanocrystals: CNCs are produced through acid hydrolysis and have high crystallinity, strength, and rigidity, which enhance the structural integrity of sensors [9].
- Cellulose nanofibers: CNFs are made using mechanical or enzymatic methods and offer flexibility and a high aspect ratio, which are important for creating reactive surface areas in sensors [10].
- Hairy nanocellulose (HNC): This form of nanocellulose is obtained by cleaving the amorphous regions in CNF, resulting in rod-shaped nanoparticles with “hairy” structures on their ends, facilitating further chemical modification, enhancing its compatibility with other materials, and increasing its accessible surface area, which is advantageous in applications like catalysis and adsorption [11]. In Figure 2, a schematic representation of cellulose structures from resources to the molecular level is depicted [12].
- Bacterial cellulose: BC is prized for its purity and unique mechanical properties. It is synthesized by bacteria without plant-based impurities such as hemicellulose and lignin [13].

The surface chemistry of nanocellulose plays a vital role in the functionality of biosensors. Its hydroxyl groups can be chemically modified, allowing for the attachment of sensing molecules or nanoparticles. HNCs can contain aldehyde, carboxyl, amine, or other groups in their hair. Moreover, utilizing nanocellulose from renewable sources highlights its environmental sustainability and its potential for creating eco-friendly biosensor designs [14].

Cellulose is a versatile raw material that can be converted into numerous products for various applications, including sensors. However, its full potential is often not realized due to challenges associated with its reduced chemical reactivity, and the limitations of cellulose reactivity are primarily due to its structural characteristics and the strong intermolecular hydrogen bonds that make it a recalcitrant polysaccharide [15].

#### 1.2.1. Reactivity Issues

Dissolution difficulty: Dissolving cellulose is important for creating high-value cellulosic products and functional materials. However, the stiffness of its chains and the numerous hydrogen bonds make it difficult to dissolve cellulose, as they prevent the chains from interacting with each other [16].

Hydroxyl group accessibility: The dissolved state of cellulose allows for the full availability of hydroxyl groups, which are essential for introducing functional groups into the cellulose structure. Nonetheless, the hierarchical structure and crystalline nature of cellulose in plant cell walls limit accessibility to these hydroxyl groups, restricting their use [12].

Chain degradation and side reactions: When chemically modifying cellulose, there is a risk of chain degradation and the occurrence of side reactions. This can reduce the yield and quality of the modified product [9].

Enzymatic hydrolysis limitations: The enzymatic hydrolysis rate of cellulose decreases with conversion, which limits its commercial use. Factors such as changes in crystallinity, accessibility, reactivity, and the hydrolysable fraction of cellulose contribute to this decline due to chemical modification [17].

#### 1.2.2. Approaches to Enhance Reactivity

Additives to improve solubility: The dissolution of cellulose in aqueous NaOH can be enhanced with additives to stabilize the solution against gelation [18].

Surface functionalization: Techniques such as TEMPO-oxidation can be used to study and quantify cellulose reactivity. Raman spectroscopy provides insights into the structural changes during functionalization by analyzing chemical bonds at the molecular level, enhancing our understanding of cellulose solubility and reactivity [12].

Linkers for functionalization: Aminopropyltriethoxysilane has been utilized as a linker for cellulose-based functional materials, offering the potential to create enhanced properties [11].

Ionic conductive hydrogels: Carboxymethyl cellulose-based ionic conductive hydrogels are used in highly sensitive, stable, and durable sensors for various applications [11].

Functionalizing cellulose with other functionalities or sensor scaffolds is indeed a challenging task due to the complex structure of cellulose and the difficulty in achieving high yields [19]. Here are some examples of attempts to functionalize cellulose and the challenges encountered:

1. Copper nanoparticles-induced trimesic acid-grafted cellulose: This approach was used to create a multifunctional textile with low chemical induction. However, the process is complex and requires careful control to ensure the successful grafting of trimesic acid onto the cellulose [19].
2. Graft copolymers of poly (ethyl acrylate) and cellulose: This approach was used to develop reactive metal ion sorbents. However, the details of the process and its challenges are not available in the search results [20].
3. Carboxymethyl cellulose (CMC) modified to have covalently tethered hydrazide groups: This approach was used to create adhesive joints between wet cellulose surfaces. However, wet adhesion was dominated by polyelectrolyte complexation, and the presence of hydrazone linkages had little influence on wet adhesion [21].

In terms of chemosensors, there are examples of chemosensors being used for the tracking of metal ions, such as the “off-on”-based fluorescent chemosensor for Cu^2+^ in aqueous media and living cells. Another example is the use of supramolecular chemosensors for enzyme assays, which can specifically signal analytes with fluorescence-based read-out methods. However, these examples do not specifically involve the functionalization of cellulose [22].

In conclusion, while there are various attempts to functionalize cellulose with other functionalities or sensor scaffolds, achieving high yields and 100% functionalization remains a significant challenge due to the complex structure of cellulose and the difficulty in controlling the grafting process.

Cellulose-based implants are gaining attention in the field of bone tissue engineering due to their biocompatibility, mechanical strength, and ability to support cell differentiation [23]. The various cellulose-based scaffold materials are summarized in Table 1, which outlines their key components, properties, and relevant references. Here are some examples of cellulose-based scaffold materials:

1. Cellulose-acetate-based composite: This composite is used as a coating for biodegradable magnesium implants. The composite includes fillers such as hydroxyapatite and magnesium particles, which have shown positive effects on biocompatibility and surface analysis [24].
2. Cellulose-based materials crosslinked with epichlorohydrin: These materials have improved thermal stability, surface area, and swelling degree of hydrogels, making them suitable for implant applications [25].
3. Wood-derived composite scaffold: This scaffold is developed from natural wood treated with an alkaline solution, resulting in a cellulose skeleton with high elasticity. The scaffold is further modified with chitosan quaternary ammonium salt (CQS) and dimethyloxalylglycine (DMOG) to enhance antibacterial, osteogenic, and angiogenic activities [26].
4. Silk fibroin/cellulose hydrogels: These hydrogels are created by dissolving silk fibroin and cellulose together, resulting in a porous structure that supports the differentiation of MC3T3 cells into osteoblasts, making them a suitable scaffold for bone tissue engineering [27].
5. Cellulose-acetate-based composite coatings on Mg3Nd alloys: These coatings are used to reduce the biodegradation rate and modify the surface properties of Mg3Nd alloys to increase osteointegration [28].
6. HDPE/Chitosan composites: These composites contain chitosan, a bio-based polymer with biocompatibility and antimicrobial properties, and are used for bone replacement applications [29].

Here is a summary Table 1 of the mentioned cellulose-based implants:

**Table 1:** Summary of Cellulose-Based Scaffold Materials and Their Properties.

Implant Type	Key Components	Key Properties	Reference
Cellulose-Acetate-Based Composite	Hydroxyapatite, Magnesium particles	Biocompatibility, Surface analysis	[24]
Cellulose-Based Materials Crosslinked with Epichlorohydrin	Epichlorohydrin	Thermal stability, Surface area, Swelling degree	[25]
Wood-Derived Composite Scaffold	Chitosan quaternary ammonium salt (CQS), Dimethyloxalylglycine (DMOG)	Antibacterial, Osteogenic, and Angiogenic activities	[26]
Silk Fibroin/Cellulose Hydrogels	Silk fibroin, Cellulose	Supports cell differentiation to osteoblasts	[27]
Cellulose Acetate-Based Composite Coatings on Mg3Nd Alloys	Cellulose acetate	Reduces biodegradation rate, Increases osteointegration	[28]
HDPE/Chitosan Composites	Chitosan	Good mechanical properties, Antimicrobial activity	[29]

### 1.3. Applications and Case Studies

The integration of 2D materials with nanocellulose has greatly expanded the application and advancement of biosensors in various fields. In this section, we will explore case studies and applications that demonstrate the practical implications and benefits of these advancements.

#### Recent Research and Advancements

Monoelemental 2D materials, such as Xenes (e.g., graphyne, silicene, germanene), have been extensively researched for their potential use in biosensors. These materials possess desirable characteristics, including high optical response capability, excellent electrical-optical properties, a large specific surface area, and easy modification of potential [30].

The utilization of cellulose, a carbohydrate biopolymer, has brought significant advancements to the field of biosensors through the development of portable paper-based biosensors and microfluidic paper-based analytical devices (μPADs) [31]. These innovative devices offer exceptional sensitivity and cost-effectiveness, making them a promising alternative to conventional advanced analytical instruments. With their applications in emergency medical situations, point-of-care health diagnostics, and early cancer screening, rPBBs and μPADs present exciting opportunities for improving healthcare outcomes [32].

Paper and cellulose-based biosensors have gained relevance during the COVID-19 pandemic due to their rapid production capabilities and suitability for use by untrained personnel. These biosensors are used in low-cost point-of-care diagnosis, including viral genomic material detection, viral antigen identification, and serological antibody testing [33].

## 2. Advantages of 2D Materials and Cellulose in Biosensors

The use of 2D materials in biosensor applications provides several advantages, including excellent mechanical, optical, and electrical properties. These properties are essential for the development of wearable biosensors that enable real-time monitoring of human health information and accurate measurement of vital signs [34]. Integrating 2D materials into wearable biosensors has expanded opportunities for early detection of life-threatening diseases and continuous health tracking. 

In addition to 2D materials, cellulose-based biosensors also offer significant benefits. They are cost-effective, highly sensitive, and compatible with portable sensing devices used in biomedical applications. One major advancement in this field is the functionalization of cellulose papers with antibodies, nucleic acids, and nanomaterials in PBBs (paper-based bioassays) and μPADs (microfluidic paper-slip devices) [34]. 

Hence, the application of 2D materials and cellulose in medical diagnostics and biosensors has shown great potential. These materials have unique properties that make them suitable for various applications, including disease detection, real-time monitoring, and point-of-care diagnostics. However, further research is needed to fully utilize their capabilities and address any challenges associated with their use. 

The use of biosensors has significantly advanced the detection of biomarkers, particularly in early cancer diagnosis. In a recent study, a biosensor incorporating graphene as a 2D material demonstrated improved sensitivity and selectivity for tumor markers. This innovative biosensor was able to detect biomarkers at concentrations as low as 1 ng/L, representing a significant advancement in early cancer detection methods [35]. 

Biosensors that incorporate nanocellulose have shown promising potential for the early detection of cancer biomarkers in blood samples. These biosensors are highly sensitive and capable of detecting biomarkers even at very low concentrations [36]. The use of 2D materials, such as nanocellulose-based materials, has also attracted significant research interest due to their unique properties, including high conductivity, hydrophilicity, and biocompatibility [37]. These properties make them suitable for various biomedical sensing applications. 

In recent developments in bioelectronics, 2D carbon networks arranged into high-order 3D nanotube arrays on flexible microelectrodes have emerged as implantable probes. These probes enable the in-situ detection of cancer tissues by differentiating them from normal tissues [38]. Their electrochemical sensing capabilities allow for the detection of H_2_O_2_ biomarkers secreted by various cancer cells and tissues—a crucial factor in cancer diagnosis and evaluation [39]. 

Moreover, electrochemical biosensors utilizing graphene and other 2D materials have shown great potential for detecting biomarkers associated with hematological malignancies like leukemia, lymphoma, and multiple myeloma. These biosensors offer a wide linear range, a low detection limit, high sensitivity, excellent selectivity, and cost-effectiveness [40]. 

In addition to that, metal nanoparticles have been employed on 2D platforms to enhance the detection of electrochemical biomarkers. This approach has demonstrated remarkable sensitivity, selectivity, fast response time, ease of use at a low cost, and simple fabrication procedures [41]. 

There have also been advancements in designing phosphorene-based nanosensors for the detection of formaldehyde as an essential lung cancer biomarker. These sensors exhibit exceptional sensitivity in detecting formaldehyde levels [42]. 

An electrochemical biosensor has been developed using reduced graphene oxide-modified and reduced-molybdenum disulfide multi-layered nanosheets for detecting neuron-specific enolase, a biomarker for lung cancer [43]. The sensor exhibits a wide linear range of detection and remains unaffected by other interfering species commonly found in human serum. 

### 2.1. Medical and Diagnostic Applications

Glucose monitoring is essential for managing health conditions such as diabetes. Recent advancements in the construction of glucose-monitoring biosensors have focused on utilizing 2D materials and cellulose [34]. However, further research is needed to enhance their performance and explore their potential in clinical practice. 

The use of 2D materials has proven beneficial in glucose sensing applications, resulting in improved sensor properties including stability, large surface area, and affordability [43]. 

Cellulose has been utilized in the development of wearable, self-powered glucose biosensors. A flexible biosensor was created using cellulose fibers coated with multi-wall carbon nanotubes and reduced graphene oxides, resulting in a porous electrode with excellent flexibility, conductivity, and electroactive surface area for urine glucose detection [34]. 

In a recent study, researchers investigated the potential of combining reduced graphene oxide (rGO) with gold nanoparticles in cellulose-nanofiber matrices to create a transducer layer for an environmentally friendly and flexible glucose sensor [39]. 

Furthermore, metal-organic frameworks such as 2D isomorphic Co/Ni-Metal-Organic Frameworks have demonstrated promise as materials for detecting glucose. These MOFs exhibit higher sensitivity, a wider linear range, and lower detection limits compared to other MOF-based glucose sensors. 

Biosensors for DNA detection are crucial in genetic testing and disease diagnosis, utilizing the high surface area of nanocellulose and the electrical conductivity of 2D materials like molybdenum disulfide [43]. A recent study showed that such biosensors achieved a sequencing error rate below 0.1% and improved sequencing speed, enhancing the precision and reliability of DNA sequencing [44]. 

Graphene-like 2D materials and cellulose were utilized to develop biosensors for DNA detection. One study investigated the interaction of a two-dimensional metal oxide with single-stranded DNA (ssDNA) as a potential method for detecting viral infections [45]. Spectroscopic measurements confirmed strong interactions between ssDNA and the metal oxide, suggesting its efficacy in detecting viral infections. Another study focused on an electrochemical biosensor using BiSbTeSe_2_, an intrinsic topological insulator, which demonstrated high sensitivity in detecting target DNA with a low detection limit of 1.07 × 10^−15^ M [41]. 

The potential of 2D materials and cellulose in biosensors is demonstrated by examples such as the development of a photoelectrochemical aptasensor for detecting SARS-CoV-2 spike glycoprotein using a two-dimensional (2D) metal-organic framework and the synthesis of TEMPO-oxidized cellulose nanocrystal-capped gold nanoparticles for colorimetric detection of unamplified pathogenic DNA oligomers. These applications show their efficacy in detecting viral infections, HIV gene determination, and SARS-CoV-2 spike glycoprotein [41,46]. 

Advanced biosensors incorporating 2D materials and nanocellulose have indeed shown promise in managing cardiovascular diseases by detecting and quantifying cholesterol. These biosensors demonstrate high sensitivity and accuracy, with a notable study achieving a detection limit of 0.1 mg/dL and a response time under 5 min [20]. The innovative design utilizes graphene oxide as a 2D material to enhance performance in clinical applications. Furthermore, cellulose-based biosensors have been successfully employed for monitoring various biomolecules, including glucose, urea, cells, amino acids, proteins, lactate, and hydroquinone [46]. 

However, while these advancements are promising, there are still areas that require further research and development. For instance, while 2D materials like graphene and MoS_2_ have been widely used in biosensors, there is a need to explore other 2D materials to discover novel properties that may contribute to the construction of high-performance electrochemical biosensors [15]. 

A critical factor in the future of electrochemical biosensing is the creation of highly sensitive biosensors through affordable and simple fabrication processes. These sensors must offer excellent sensitivity, selectivity, reliability, and stability. Cellulose-based biosensors have shown promise but may require additional chemical treatments due to their hydrophilic nature being incompatible with certain molecular sensors [47]. Moreover, there is a need for advancements in miniaturizing cholesterol and dopamine biosensors for lab-on-chip devices to overcome existing technical limitations and enable convenient use by patients at home [45].

In conclusion, while advanced biosensors incorporating 2D materials have shown significant promise in managing cardiovascular diseases, there are still challenges to be addressed and potential directions for future research. These include exploring different 2D materials, enhancing fabrication procedures, addressing the hydrophilic nature of cellulose, and improving biosensor miniaturization. 

Cellulose-based biosensors have also been developed specifically for glucose and urea detection. Their accurate readings enable effective management of conditions such as diabetes or kidney disease. These sensors employ immobilized enzymes on a cellulose substrate to generate an electrical signal proportional to the concentration of glucose or urea [46]. 

Cellulose-based biosensors have found applications in the detection of specific diseases by identifying cells and proteins associated with certain conditions. In cancer diagnostics, they can detect specific cancer-associated proteins or cancer cells, enabling early disease diagnosis and monitoring [46]. 

Furthermore, cellulose-based biosensors have shown usefulness in pathogen detection for diagnosing infectious diseases. By detecting specific biomarkers associated with pathogens, these biosensors facilitate rapid and accurate diagnoses [48]. 

### 2.2. Environmental Monitoring

Water quality analysis: Graphene-based biosensors offer a potential solution for real-time environmental monitoring and pollution control by detecting trace amounts of heavy metals in water sources. Graphene and other 2D materials have been studied for their potential in analyzing and treating water quality. Graphene, especially graphene nanoflakes, has been used to create hydrogels for biosensors, showing promise as a material for water quality analysis. Furthermore, graphene microfiber membranes have been developed to remove nano-sized pollutants from water. The effectiveness of these membranes has been improved through surface functionalization that adjusts the interlayer separation in 2D membranes, enhancing their sieving capabilities. The combination of graphene oxide and cellulose acetate creates a unique adsorbent for efficient phosphate removal from water [49]. The presence of hydroxyl groups on the modified surface of cellulose acetate aids in the process [50]. Moreover, field-effect transistors made from 2D materials find application in water-related sensing. For instance, MoS_2_ nanosheets can be inkjet-printed into ultrathin semiconducting channels to fabricate FET-based water sensors. These sensors demonstrate high selectivity towards Pb2+ even at low concentrations (as low as 2 ppb), highlighting the potential use of 2D materials in assessing water quality. 

Cellulose and 2D materials have displayed promising potential in water quality analysis, treatment, pollutant removal, biosensing, and monitoring. These filters can capture specific aquatic impurities for which antibodies can be produced and attached to the filters [51].

The utilization of two-dimensional nuclear magnetic resonance (2D NMR) techniques has allowed for the qualitative analysis of substructures of water-soluble organic compounds in atmospheric aerosols. This includes identifying spectral signatures associated with anhydrosugars present in cellulose. In terms of air quality monitoring, both 2D materials and cellulose offer high sensitivity for detection. A study demonstrated the use of a sensor composed of reduced graphene oxide (rGO) and graphene oxide on a cellulose-acetate composite membrane, which exhibited excellent sensitivity to minute changes in air quality. Furthermore, one notable advantage is their flexibility and adaptability for various applications. The same study emphasized that the film’s flexibility makes it suitable for wearable electronic devices or other applications that require flexible substrates. 

Recent research has investigated the utilization of cellulose-based materials for analyzing indoor air quality. These innovative thermal insulation materials, obtained from recycled cellulosic and/or animal waste, were thoroughly examined [52]. 

Another study focused on developing a highly sensitive, flexible sensor. The sensor was created using an electro-spun composite membrane with abundant mesopores. This sensor exhibited exceptional sensitivity at low strains, making it ideal for potential use in wearable electronic devices. 

In a separate investigation, researchers studied the effectiveness of composite membranes containing a 2D material and carbon nanotubes in solar steam generation and desalination processes. These composite membranes demonstrated self-floating capabilities on the air-water interface and minimized the accumulation of salt during de-salination due to their hydrophobic nature [53]. 

The application of 2D materials and cellulose In air quality monitoring exhibits great potential due to their flexibility and sensitivity. Recent studies have demonstrated their versatility and innovative possibilities in various fields, including thermal insulation and wearable electronic devices [54]. The application of 2D materials and cellulose in various fields, including thermal insulation, wearable electronic devices, and air quality monitoring, indeed exhibits great potential due to their flexibility and sensitivity. However, several challenges need to be addressed to fully realize their potential. 

A major hurdle in developing wearable electronic devices using 2D materials and cellulose is finding the right balance between mechanical stretchability and electronic performance. For example, stretchable electronics, which are crucial for wearable devices, require substrates that can withstand stretching while maintaining their electronic functionality. PDMS is commonly used due to its ease of fabrication and low cost; however, enhancing its mechanical stretchability without compromising electronic performance remains a significant challenge [55].

The fabrication methods also present considerable challenges. Modern electronic devices are designed for 2D planar substrates, which are generally ill-suited for the intricate 3D structures of textiles. Consequently, there is an urgent need to develop fabrication techniques specifically tailored for e-textiles to advance wearable electronics [56].

The development of efficient, flexible, and scalable energy storage solutions remains a significant challenge for powering wearable electronic textiles. Additionally, technical breakthroughs are needed for manufacturing state-of-the-art 2D layered nanomaterial-supported flexible/stretchable sensors and power devices, which are crucial to the development of wearable biomedical sensors and power devices [57]. Lastly, strain engineering presents opportunities and challenges for creating flexible nanoelectronics and optoelectronic devices. Most of these devices utilize chemical vapor deposition or mechanical exfoliation of ultrathin 2D TMD materials, which may limit their application in wearable and implantable devices with higher train tolerance [58].

In summary, while 2D materials offer promising opportunities in various applications, there are significant challenges related to mechanical stretchability, electronic performance, fabrication methods, energy storage, and strain engineering that need to be addressed to advance this field.

### 2.3. Food Safety

Pathogen Detection in Food Products: One approach involves the use of enzyme-based paper biosensors for monitoring food freshness and predicting spoilage. For instance, a biosensor has been developed to measure the release of hypoxanthine, an indicator of meat and fish degradation. This is accomplished through enzymatic conversion facilitated by XOD within a sol-gel biohybrid on paper that retains the reaction products [59]. 

The unique optical properties of 2D materials like graphene and transition metal dichalcogenides enhance biomolecule detection in optical biosensors. These materials can be modified to improve sensitivity and detection limits and are commonly used alongside techniques such as surface plasmon resonance, fluorescence resonance energy transfer, and evanescent waves for detecting biomolecules. Nanomaterials like carbon nanotubes, magnetic nanoparticles, gold nanoparticles, dendrimers, graphene nanomaterials, and quantum dots are widely used in biosensors due to their unique properties. One application is the use of fiber-optic surface plasmon resonance sensors based on 2D materials for detecting different types of cells by measuring peak power loss changes [60]. 

Intelligent packaging technology is emerging to directly monitor food quality, eliminating the need for complex processes. This can be leveraged to detect microorganisms that cause foodborne illnesses visible on the food package itself [61]. 

Achieving high-quality sensing performance, including sensitivity and stability, relies heavily on maintaining stable biosensor interfaces. To improve interface properties, nanomaterials like chitosan and cellulose are often combined with polymers [62]. 

In order to detect pathogens in food products, researchers are employing a combination of 2D materials and cellulose-based biosensors. By integrating different materials, methods, and technologies with their respective advantages and challenges, this approach aims to enhance the sensitivity and stability of these biosensors. 

### 2.4. Agricultural Applications

Soil Health Monitoring: Incorporating nanocellulose and 2D materials into biosensors allows for monitoring soil nutrients and pH levels to support sustainable farming practices. The use of these materials in sensor technology has been explored in various contexts, such as health monitoring and environmental sensing, but there is limited documentation on their specific application for soil health monitoring [63]. 

### 2.5. Health Monitoring 

Cellulose-based sensors have been developed for health monitoring purposes. A plantar wearable pressure sensor utilizing hybrid lead zirconate-titanate/microfibrillated cellulose piezoelectric composite films has been proposed for measuring plantar pressure. Similarly, a high-performance humidity sensor based on GO/ZnO/plant cellulose film has been developed for respiratory monitoring [64,65]. 

The utilization of 2D materials in health monitoring wearable devices is another area where they have found applications. Carbon black and MoS_2_, as 1D and 2D nanomaterials, respectively, have been extensively studied as core sensing components for flexible strain sensors that are used to monitor the vital signs of patients [66,67]. Furthermore, 2D materials are being employed in enzymatic and nonenzymatic glucose sensing applications. Additionally, a GaSe-based fiber optic sensor has been specifically designed for lactate sensing in human sweat as an indicator of a potential heart attack [68]. 

Regarding soil health monitoring sensors, the search results provided limited evidence on the specific utilization of 2D materials and cellulose. Although there is a wireless subsoil health sensor developed for detecting volumetric water content, it does not make use of these materials. Further research is required to explore their potential in soil health monitoring applications. 

Wearable health devices offer continuous health monitoring through the integration of flexible and biocompatible biosensors. These biosensors, composed of 2D materials and cellulose derivatives, can track vital health parameters such as heart rate and blood oxygen levels in real-time [69]. 

Graphene and other 2D materials have seen extensive use in developing wearable biosensors, which can be integrated into various wearable platforms like wristbands, headbands, and smart garments. These materials offer exceptional mechanical, optical, and electrical properties that make them ideal for such applications [70]. Additionally, certain advancements have been made in utilizing cellulose-a biomass material, in the fabrication of wearable biosensor devices [71]. Most notably, nanocellulose’s high specific surface area, biodegradability, cost-effectiveness, and sustainability are key factors behind these developments. For instance, a self-powered glucose biosensor was created from a flexible textile matrix made of cellulose fibers [72]. The sensor featured a porous three-dimensional electrode with excellent flexibility, surface conductivity, and electroactive surface area achieved by coating multi-wall carbon nanotubes and reduced graphene oxides onto the cellulosic textile matrix [73]. 

Wearable biosensors made from 2D materials and cellulose have gained attention in health monitoring for providing real-time data on vital signs. They are utilized in glucose monitoring, biomarker detection, and remote healthcare monitoring. These biosensors provide a versatile approach to personalized healthcare delivery with their design, fabrication, performance, sensitivity, and various health monitoring applications [74]. These materials offer flexibility, wearability, and responsiveness to external stimuli, which makes them well-suited for wearable health monitoring applications [75]. For example, a smart fabric based on MXene can be created by depositing Ti_3_C_2_Tx nanosheets onto cellulose fiber non-woven fabric. This fabric has a sensitive and reversible response to humidity, making it suitable for wearable respiration monitoring applications. It also has the potential to be used as a low-voltage thermotherapy platform due to its fast and stable electro-thermal response [76,77]. Furthermore, cellulose nanofibrils and Ti_3_C_2_ MXene can be combined through 3D printing to create smart fibers and textiles with responsiveness to various external stimuli. These materials have shown potential as strain sensors [68]. 

In addition, a plant-based substrate called “sporosubstrate” has been developed using natural pollen that is non-allergenic. This substrate allows for the creation of flexible shapes with customizable properties and performance characteristics. It finds application in electronic healthcare devices and wearable wireless heating systems [78,79]. 

Flexible and lightweight biosensors have been developed using 2D graphene oxide and Ti_3_C_2_ nanosheet-based supercapacitors. These sensors exhibit high sensitivity and a wide detection range and can be integrated into wearable monitoring systems for tracking physical status during various activities. The use of a cellulose-blend cloth substrate further enhances the versatility of these biosensors in wearable health applications [80]. 

### 2.6. Integration of 2D Materials and Nanocellulose in Biosensor Technologies 

Integrating 2D materials with cellulose in biosensors enhances their properties, such as sensitivity, stability, and flexibility. Graphene and transition metal dichalcogenides like MoS_2_ and WS_2_ have unique physical properties that improve the performance of biosensors. The electrical conductivity and mechanical strength of graphene enhance sensitivity and specificity, while the layered structure of transition metal dichalcogenides provides precise electrical characteristics for biosensor applications [81]. 

Combining 2D materials with nanocellulose improves biosensor performance by leveraging the high surface area of nanocellulose and the electrical properties offered by 2D materials. This combination results in highly sensitive sensors. Additionally, incorporating cellulose into 2D materials enhances the mechanical strength, biocompatibility, and environmental stability of biosensors [82]. The integration of graphene and molybdenum disulfide with cellulose creates flexible and stretchable electronic devices suitable for wearable electronics and sensors. The biocompatibility of nanocellulose contributes positively to biosensor functionality, while its mechanical strength enhances stability [83]. 

Integrating cellulose with 2D materials in biosensors enhances device strength, biocompatibility, and environmental stability. This integration is driving research opportunities and expanding the applications in this rapidly advancing field. 

The most common properties tested in biosensors include:

1. Electrical properties: These materials are significant contributors to the performance of biosensors. Graphene, with its high electrical conductivity achieved through wet-chemical methods, is essential for device performance. Similarly, materials like MoS_2_ and WS_2_ possess exceptional electrical properties due to their strong covalent and van der Waals interactions. Notably, the covalent functionalization of these atomic-thin materials with a large surface-to-volume ratio is critical to optimizing biosensor functionality [84]. Graphene’s high electrical conductivity, achievable through wet-chemical methods, is integral to device performance. MoS_2_ and WS_2_, with their strong covalent and van der Waals interactions, exhibit notable electrical properties. The covalent functionalization of these materials is vital due to their atomic thinness and large surface-to-volume ratio [85].
2. Mechanical properties: Methods such as mechanical exfoliation and chemical vapor deposition yield 2D materials with distinct mechanical characteristics, which play a crucial role in biosensing applications. Techniques like mechanical exfoliation and chemical vapor deposition enable the production of 2D materials with unique mechanical attributes for biosensor applications, thanks to their atomic thinness and extensive surface area [86].
3. Chemical properties: To enhance the functionality of biosensors, it is important to consider the chemical properties of nanocellulose. Nanocellulose contains hydroxyl groups that can be chemically modified, allowing for the attachment of sensing molecules or nanoparticles [87].
4. Optical properties: The optical properties of 2D materials such as graphene and transition metal dichalcogenides improve biomolecule detection in optical biosensors. These materials can be modified to enhance sensitivity and detection limits [88].
5. Thermal properties: In addition to other properties, certain biosensors also measure thermal conductivity. For instance, a study examined the heat transfer capabilities of polypyrrole/carbon black composite-coated cellulose (cotton) yarn. The objective was to gain insights into the behavior and performance of these materials in terms of their ability to conduct heat [89].
6. Sensitivity and specificity: The use of graphene and transition metal dichalcogenides in biosensors has significantly improved their sensitivity and specificity. These materials have a large surface-to-volume ratio, allowing for better interaction with analytes and enhanced detection capabilities [90]. Biosensors employing 2D materials and cellulose have shown increased sensitivity and specificity in applications such as early cancer diagnosis, biomarker detection, glucose monitoring, and DNA detection. These materials demonstrate exceptional electrical properties and high surface area, contributing to their enhanced performance [91].
7. Stability: The mechanical strength of nanocellulose significantly enhances the stability and durability of biosensors. This improvement is primarily due to several key properties of nanocellulose:
  - High Surface Area
  - These characteristics of nanocellulose facilitate greater enzyme immobilization in biosensors. This is crucial because it allows for higher protein loadings, which in turn improves the sensor’s response and stability [92].
  - High Young’s Modulus: This property indicates that nanocellulose is a stiff material. In the context of biosensors, a higher Young’s modulus translates to improved structural integrity, making the biosensors less prone to deformation under stress [93].
  - Biodegradability and renewable nature: These environmentally friendly properties of nanocellulose make it an ideal material for sustainable biosensor development [94].
  - Tunable surface chemistry: The ability to modify the surface of nanocellulose allows for better integration with other materials in biosensors, potentially leading to enhanced performance and specificity [95].
  - Synergistic effects with other materials: When combined with other materials, like in the case of rice starch-based edible films, nanocellulose not only enhances mechanical strength but also can impart other desirable properties to the composite material. This synergy can be leveraged in biosensors to create more robust and efficient devices [96].

In summary, the incorporation of nanocellulose into biosensors offers a multitude of benefits, primarily due to its strong mechanical properties, high surface area, and customizable nature. These characteristics contribute to the development of more stable, durable, and potentially more sensitive biosensing devices.

8. Flexibility: Combining the unique properties of nanocellulose with flexible and durable 2D materials makes them an ideal choice for developing wearable biosensors suitable for real-time health monitoring [97].
9. Cost-effectiveness: Portable paper-based biosensors and microfluidic paper-based analytical devices (μPADs) are cellulose-based biosensors that offer high sensitivity and affordability. These innovative devices provide a viable alternative to conventional advanced analytical instruments [98].
10. Environmental sustainability: By utilizing nanocellulose from renewable sources, the environmental sustainability of biosensors can be emphasized while also highlighting their potential for being eco-friendly as products and technologies [99].

In cellulose nano-whisker/graphene nano-platelet composite films, the sensing capabilities of a biosensor depend on its design and functionalization [100]. Biosensors can be designed to detect substances such as glucose, cholesterol, lactate, DNA, RNA, proteins, and various types of cells through a reaction with a specific enzyme or molecule that is attached to the sensor, causing a detectable change in the sensor’s properties [101].

Graphene and its hybrid materials are currently utilized in the development of high-performance electrochemical biosensors. They serve as advanced electrodes due to their large accessible surface area, electrical conductivity, and capacity for immobilizing enzymes [102]. For example, a glucose biosensor can be functionalized with glucose oxidase, an enzyme that reacts with glucose to produce hydrogen peroxide and gluconolactone. The hydrogen peroxide can then be detected electrochemically. Similarly, a DNA biosensor might be functionalized with a specific sequence of DNA that binds to the target DNA sequence, which causes a detectable change in the sensor’s properties [103].

Composite materials, such as the ones mentioned, have been extensively researched in various fields, including wearable electronics, energy storage, sensing devices, and environmental monitoring. Wearable biosensors made from 2D materials and cellulose have demonstrated potential in health monitoring by collecting real-time data on vital signs, glucose levels, biomarker detection, and remote healthcare monitoring [104].

### 2.7. Biosensor Functionality

The electrical and mechanical properties of these advanced materials significantly contribute to biosensor functionality. For instance, in plasmonic biosensing and photoelectrochemical (PEC) biosensing, the sensitivity of biosensors is notably enhanced by 2D nanomaterials [105].

### 2.8. Comparison with Other Technologies

Biosensors utilizing 2D materials and cellulose offer numerous advantages compared to traditional biosensors and other advanced analytical instruments. These include unique electrical and optical properties, high specific surface area, excellent mechanical properties, good thermal and chemical stability, high catalytic activities, facile synthesis process, and large surface areas for toxin detection improvement with increased selectivity and sensitivity due to their outstanding electrical signal amplification capabilities [106].

The performance of these biosensors can vary in terms of sensitivity, specificity, and stability compared to traditional biosensors and other advanced analytical instruments. Further research is needed to optimize their performance and address any associated challenges with their use.

### 2.9. Theoretical Foundations

The theoretical foundations related to the integration of 2D materials with cellulose in biosensors and other applications primarily revolve around the principles of quantum mechanics, band structure engineering, and strain engineering.

- Band structure engineering: Band theory, an approximation to the quantum state of solids, has been instrumental in the development of modern integrated solid-state electronics. The emerging 2D layered materials, with their unique electrical, magnetic, optical, and structural properties, provide a platform to implement quantum-engineered devices. These materials allow for the exploration of advanced quantum mechanical effects, such as band-to-band tunneling, spin–orbit coupling, spin–valley locking, and quantum entanglement, which are crucial for energy-efficient electronics and optoelectronics [107]. Band structure engineering is a key theoretical foundation for manipulating the electronic properties of 2D materials. This involves modifying the energy bands of these materials to control their electronic and optical properties. Techniques for band structure engineering include localized chemical doping, dual gating, liquid gating, thickness modulation, and constructing heterojunctions [108].
- Strain engineering: Strain engineering is a promising approach to tuning the electrical, electrochemical, magnetic, and optical properties of 2D materials. This involves applying mechanical strain to these materials to alter their properties, a technique that has the potential for high-performance 2D-material-based devices. Strain engineering can fundamentally change the electronic and optoelectronic properties of 2D materials, leading to novel functional device applications [109,110].

These theoretical foundations provide the basis for understanding and manipulating the properties of 2D materials when integrated with cellulose, thereby enhancing their performance in various applications, including biosensors.

The incorporation of 2D materials into biosensor technology significantly enhances their performance by leveraging various physical properties at the nanoscale. Specific examples include graphene and molybdenum disulfide, which exhibit exceptional physical, mechanical, and optical characteristics that render them ideal for nanoelectronic devices and sensors [111]. These materials can be combined with cellulose-based compounds to form blends with enhanced attributes for applications in energy storage, optoelectronics, and biological control. By integrating nano-sized substances into Na-CMC blends through strain engineering techniques, it becomes possible to manipulate the band structure of 2D materials for continuous tuning purposes [112,113]. Consequently, this integration facilitates the development of high-performance biosensors characterized by improved sensitivity, selectivity, and stability.

High surface-to-volume ratios: The large surface-to-volume ratio of 2D materials offers significant advantages in various applications, including adsorption, sensing, and catalysis. These materials have shown potential as effective adsorbents for environmental decontamination due to their high surface area, specific binding capability, and chemical stability [114].

Combining 2D materials with cellulose can further enhance their properties. For example, graphene oxide is used to strengthen cellulose nanofibrils in the production of CNF-GO nanocomposite films, resulting in improved organization of the CNF matrix. Additionally, MXene-cellulose nanofiber composites have been developed for applications such as electromagnetic interference shielding materials [115].

The high surface-to-volume ratio of cellulose-based 2D materials contributes to their mechanical properties. Mistletoe viscin, a natural adhesive made up of hierarchically organized cellulose microfibrils, can be transformed into stiff and sticky fibers, showcasing its potential for bioinspired and biomedical uses [116,117].

Additionally, 2D materials such as MoS_2_ and WS_2_ exhibit enhanced electrical properties due to their high surface-to-volume ratio. These materials can replace silicon in transistors because they can drive high currents with low leakage currents and possess nonvolatile switching characteristics [118]. Therefore, integrating cellulose with 2D materials expands their applications in environmental decontamination, sensing, catalysis, and electronics.

### 2.10. Electrical Conductivity and Electron Mobility

The integration of cellulose with 2D materials has demonstrated promising potential for improving electrical conductivity and electron mobility across various applications, including wearable electronics, energy storage, and sensing devices. For example, the use of cellulose-based ion gels in fabricating aerosol-jet-printed electrolyte-gated indium oxide thin-film transistors resulted in devices with electron densities surpassing 7 × 1014 cm^−2^ and mobilities exceeding 11 cm^2^ V^−1^ s^−1^ [119]. Furthermore, stretchable thermal sensors for wearable electronics were developed using polypyrrole-coated threads that maintained consistent electrical conductivity even under tensile strains above 100% [120]. These instances emphasize the advantageous prospects of combining cellulose with 2D materials.

Mechanical properties: Integration of 2D materials with cellulose has been found to enhance the stability and durability of biosensors, offering the potential for functionalization and integration with various biomolecules. The mechanical properties of cellulose integrated with specific 2D materials are influenced by the fabrication process and modifications applied [121]. Research has shown that graphene and molybdenum disulfide can be incorporated into cellulose to create flexible and stretchable electronic devices suitable for wearable electronics and sensors. For example, a flexible capacitor was fabricated using a few-layer MoS_2_ grown on aluminum foil as electrodes and cellulose paper as a dielectric material, demonstrating enhanced capacitance upon strain due to its piezoelectric property.

Various approaches have been used to study the tensile properties of all-cellulose composites. The assessment of tensile properties can be done using nominal stress and strain equations, considering factors such as force, cross-section area, displacement, and initial length [122].

A study demonstrated that modifying the surface properties of natural cellulose fibers through chemical methods improves the mechanical properties of graphene/cellulose conductive paper. The study found that the carboxyl content in cellulose had a significant impact on the mechanical properties of graphene/oxidized-cellulose conductive paper. When the carboxyl content was low, graphene/oxidized-cellulose conductive paper achieved an elastic modulus value of 1572 MPa, which was 27.4% higher compared to cellulose/graphene conductive paper [123,124].

Cellulose, a readily available and cost-effective natural material, offers impressive mechanical properties and structural stability. It has the potential to replace carbon fiber/epoxy composites with cellulose/epoxy composites that deliver excellent performance at an affordable price. Nanolization of cellulose is crucial for ensuring composite strength, while the formation of continuous macroscopic structures ensures high Young’s modulus in these composites [125].

In summary, the integration of 2D materials with cellulose presents promising prospects for various applications, particularly in flexible and wearable electronics. However, specific properties may vary depending on the choice of 2D materials used, fabrication processes employed, and modifications applied to cellulose.

### 2.11. Environmental and Economic Aspects

The environmental cost as well as the economic cost are factors of major influence when establishing the acceptance of technology. The environmental cost includes aspects such as toxicity of the extraction process, fabrication, usage, and disposal. Cellulose provides great benefits in all the above categories. Below, some of the environmental and economic-related aspects are discussed.

### 2.12. Environmental Aspects

2D materials, including graphene and other 2D metal-organic frameworks, can be synthesized in a cost-effective and environmentally friendly manner. These materials have great potential for use in advanced electrical devices and integrated circuits. Cellulose, an abundant renewable resource on earth, serves as the foundation for these 2D materials. It possesses excellent properties such as biocompatibility, environmental friendliness, and chemical stability [126].

In the field of all-solid-state flexible supercapacitors, cellulose-based hybrid 2D material aerogels have been utilized. These aerogels are created through a supercritical CO_2_ drying process using cellulose nanofibers to effectively disperse other 2D materials like molybdenum disulfide and reduced graphene oxide [127].

Additionally, cellulose has found utility in environmental applications such as industrial water decontamination. Its efficacy and affordability make it a desirable choice for this purpose. Selective oxidation of cellulose has been employed to produce novel high-performance materials with diverse applications in fields including bio-medical engineering, healthcare, energy storage, barriers, sensing technologies, and food packaging [128].

### 2.13. Economic Aspects

Combining 2D materials with cellulose provides notable economic advantages. This integration enables the development of self-powered sensors for various applications, such as biomedicine, environmental detection, human motion monitoring, energy harvesting, and smart wearable devices. Additionally, combining cellulose with MXene results in the cost-effective manufacturing of green and highly efficient electromagnetic interference shielding materials. Furthermore, it allows for the production of all-solid-state flexible supercapacitors using cellulose-based hybrid 2D material aerogels [127,128,129].

To conclude, integrating 2D materials into cellulose offers significant environmental and economic benefits. This makes it a highly promising field for future research and development efforts.

## 3. Examples and Discussion

The unique electrical, mechanical, and chemical characteristics of two-dimensional (2D) materials and nanocellulose make them valuable in enhancing biosensor functionality across various domains in the field of biosensor technology.

### 3.1. Enhanced Sensitivity and Specificity

The utilization of 2D materials like graphene and transition metal dichalcogenides in biosensors has significantly enhanced their sensitivity and specificity. These materials possess a large surface-to-volume ratio, facilitating better interaction with analytes and improving detection capabilities. Graphene-based biosensors have demonstrated remarkable sensitivity in detecting glucose levels, which is advantageous for managing diabetes. Similarly, sensors based on MoS_2_ exhibit increased specificity in differentiating various biomolecules, making them essential for analyzing complex biological samples [130].

### 3.2. Wearable and Flexible Biosensors

**Figure 3:**
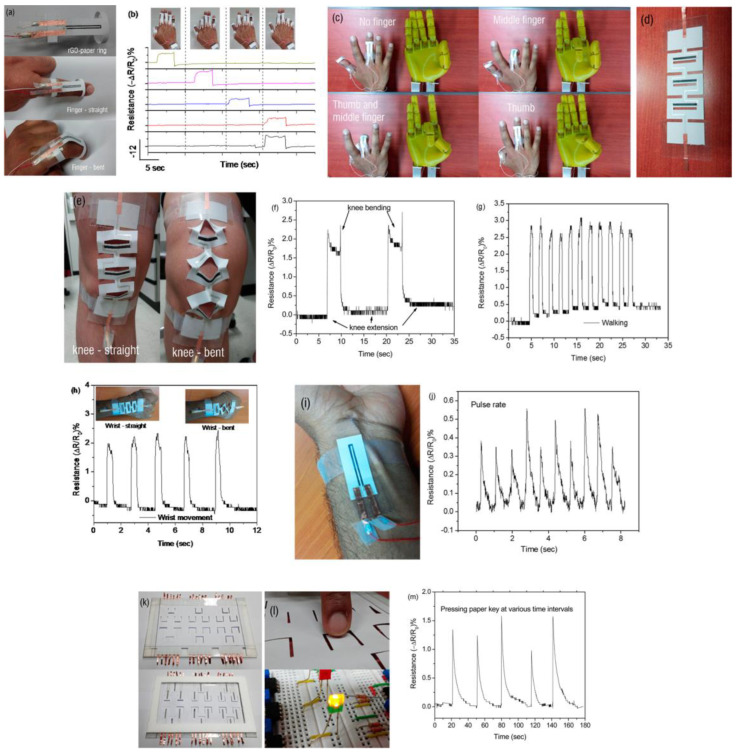
The rGO-paper rings demonstrate flexibility in different positions. (**a**) Photos show an rGO-paper ring in extended and bent positions. (**b**) Response signals from five independent rGO-paper rings monitoring various finger positions are depicted. Photographs of the hand in four different positions, corresponding to the plotted signals, are included. (**c**) The middle finger and thumb of a 3D-printed robotic hand can be controlled using rGO-paper rings. (**d**) An intricate kirigami pattern is created through a multilayer masking process using rGO-paper. (**e**) Positions while wearing rGO-paper sensors. Observing changes in the resistance of an rGO-paper sensor on a knee during (**f**) sitting and (**g**) walking. (**h**) Response curve from wrist movement. (**i**) rGO paper sensor for pulse detection on the wrist, (**j**) and its corresponding signal data display. (**k**) Laminated paper keyboard with images of both sides (top and bottom view). (**l**,**m**) By touching a paper key, an LED illuminates and generates the corresponding signal. Reproduced with permission from [131].

The combination of flexible and strong 2D materials with the lightweight and biocompatible nature of nanocellulose makes them ideal for wearable biosensors. These materials allow for the creation of sensors that can conform to the body, providing continuous monitoring of health without causing discomfort. For instance, a skin patch sensor can track sweat composition to provide insights into hydration levels and electrolyte balance. This application demonstrates how material properties directly impact user comfort and sensor effectiveness. Figure 3a illustrates an overview of the rGO-paper ring in different positions on a bent or extended finger, with the inner ring securely attached to the knuckle while allowing flexibility for bending or straightening along with finger movements. A few remarkable applications of cellulose and 2D materials are presented below.

Figure 3a depicts the rGO-paper ring and its placement on a bent and extended finger. These wearable sensors are made from reduced graphene oxide (rGO) arranged on paper substrates, making them highly responsive to deformations such as bending and folding. and Figure 3b shows the response of rGO-paper rings when various fingers are bent or extended. In a practical application shown in Figure 3c, real-time control of a 3D-printed robotic hand is demonstrated using intelligent rGO-paper rings. The changes in compressive and tensile resistances of these rings serve as inputs to control specific motions, such as those performed by the middle finger and thumb.

To detect different human motions involving knee and wrist joints with skin stretching up to 55%, intricately designed rGO-paper strain sensors were prepared. These sensors were made using flexible paper substrates and a precise digital craft cutter. They featured parallel rGO lines and conductive silver connections patterned on a stretchable paper kirigami through the multilayer masking method. The sensor was designed with alternating concave and convex bends in the paper strips to ensure that the strain-sensitive rGO patterns remained effective. Silver lines were used to connect these patterns (Figure 3d). When the knee or wrist sensor is bent, the rGO-patterned paper strips produce positive or negative signals depending on the direction of bending. The response behavior of this wearable pressure sensor was demonstrated for various motions such as knee bending, walking, and wrist bending (Figure 3e–h). These sensors also allow for real-time pulse reading, as shown in Figure 3i,j. Wearable electronics that do not hinder body movement have gained attention due to their versatility across multiple applications. In addition, rGO-paper sensors are highly sensitive to folding and bending while also being easy to manufacture. This allows for greater freedom in creative design within a wide sensing range. One potential application of incorporating rGO-paper sensors is in paper keyboards, where they can effortlessly detect when keys are touched. The detected signals can then be processed according to predefined instructions. To demonstrate the feasibility of this concept, we used the multilayer masking method to create U-shaped rGO patterns and silver connecting lines on paper cantilevers. Figure 3k shows both the top and bottom sides of an rGO-paper keyboard, with various LEDs illuminated by utilizing the signals from individual keys as shown in Figure 3m. To detect a wide range of human motions involving skin stretching up to 55% in knee and wrist joints, they fabricated intricately designed rGO-paper strain sensors on flexible paper substrates using a digital craft cutter. Using the multilayer masking technique, parallel rGO lines and conductive silver connections were patterned onto a stretchable paper kirigami. This design allowed for proper detection and differentiation of various motions by selectively patterning strain-sensitive rGO on specific paper strips, preventing signal nullification caused by opposite bending directions. The interconnected rGO patterns were joined by silver lines (Figure 3d), generating positive or negative signals based on the direction of bending as the knee or wrist is stretched uniformly by all the rGO-patterned paper strips. These rGO-paper sensors have proven to be effective in detecting and distinguishing various movements such as knee bending, walking, and wrist flexion (Figure 3e–h). They are also capable of accurately measuring pulse rates in real-time (Figure 3i,j). Wearable electronics that can monitor body motion without impeding movement have gained significant attention across a wide range of applications. The high sensitivity to bending and folding exhibited by rGO-paper sensors makes them ideal for fabrication purposes. Additionally, their versatility allows for the development of innovative designs for various sensing applications. Apart from being used as wearable electronics, the remarkable sensitivity of these sensors makes them suitable for creating paper keyboards that can detect keytouches. The detected signals can then be processed according to specific instructions.

To validate this concept, U-shaped reduced graphene oxide (rGO) patterns and silver connecting lines were patterned on paper cantilevers using a multilayer masking technique. The design of an rGO-paper keyboard is showcased in Figure 3k, which shows both the top and bottom sides. Figure 3m demonstrates how individual keys are used to illuminate different LEDs accordingly [131].

### 3.3. Environmental Monitoring Applications

The high sensitivity of materials like graphene oxide makes them valuable in environmental monitoring. They can detect minute chemical changes and trace amounts of heavy metals and pollutants in water, showcasing their potential to address global environmental challenges [132].

**Figure 4:**
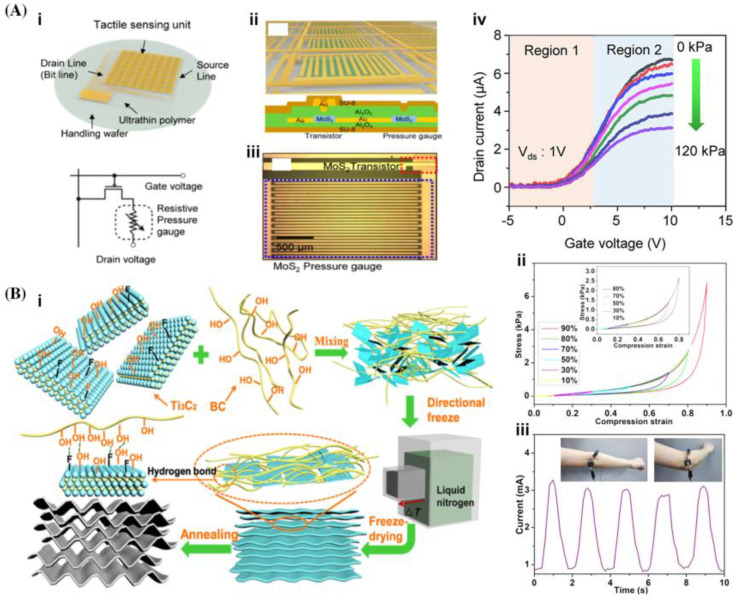
(**A**) (**i**–**iii**) The schematic structure and images of the MoS2 sensor. (**iv**) Pressure-transfer parameters of the MoS2 sensor from 0 to 120 kPa. (**B**) (**i**) Illustration of fabricating C-MX/BC-x carbon aerogel, where C, MX, BC, and x represent carbonization, Ti3C2, bacterial cellulose, and the mass ratio of BC to Ti3C2, respectively. (**ii**) Stress–strain curves at various compression strains with current signals from (**iii**) elbows. Reproduced with permission from [133].

In addition to monitoring human physiological signals, 2D materials can also be used for visualizing other physical signals such as pressure and strain. Pressure sensors based on piezoresistive, capacitive, and triboelectric effects have been developed using various types of 2D materials. The integration of 2D MoS_2_ into high-k Al_2_O_3_ layers has enabled a large-scale tactile sensor to effectively detect pressures ranging from 1 to 120 kPa. This sensor exhibits high sensitivity (ΔR/R0: 0.011 kPa^−1^) and a fast response time (180 ms), as illustrated in Figure 4A(i–iv), addressing the interference challenges associated with traditional passive pressure sensors. These findings imply potential applications in multitouch perception and handwriting recognition. Furthermore, previous studies have highlighted the piezoresistive properties of compressible and flexible carbon aerogels incorporating carbon nanotubes, graphene, and other 2D nanomaterials. These materials are well-suited for use in wearable bioelectronics and electronic skins to detect tactile contacts with exceptional sensitivity. To enhance the interaction between nanomaterials and improve the performance of sensors, a solution is to use bacterial cellulose as a binding agent. By connecting Ti_3_C_2_ nanosheets, lightweight CECAs (Compressible and elastic carbon aerogels) can be created for electronic devices. These CECAs exhibit continuous lamellae with wave-like or oriented patterns that provide excellent compression properties, maintaining elasticity even under strains up to 99% for over 10,000 cycles (Figure 4B(ii,iii)). The results demonstrate that this device displays remarkable sensitivity across a wide range of pressures, from 0 kPa to 10 kPa, with exceptional linearity up to a strain of 95%. Additionally, it exhibits increased sensitivity to small changes in strain and pressure conditions [133].

### 3.4. Medical and Diagnostic Applications

The use of biosensors made with 2D materials has greatly advanced biomarker detection in the medical field. These sensors have transformed the development of efficient diagnostic tools, allowing for accurate and rapid detection of cardiac biomarkers from small blood samples [134]. This enables prompt diagnosis in emergencies. Additionally, these biosensors are biocompatible and minimize the risk of adverse reactions, making them suitable for long-term implantable devices [135].

The regulation of glucose levels is crucial to managing diabetes on a global scale. To meet the growing demand for affordable and versatile sensing systems, an amperometric sensor was proposed to detect glucose using patterns of reduced graphene oxide (rGO). The method involves depositing patterned graphene oxide on substrates through filtration techniques such as stamping, followed by reducing it to rGO to ensure conductivity.

**Figure 5:**
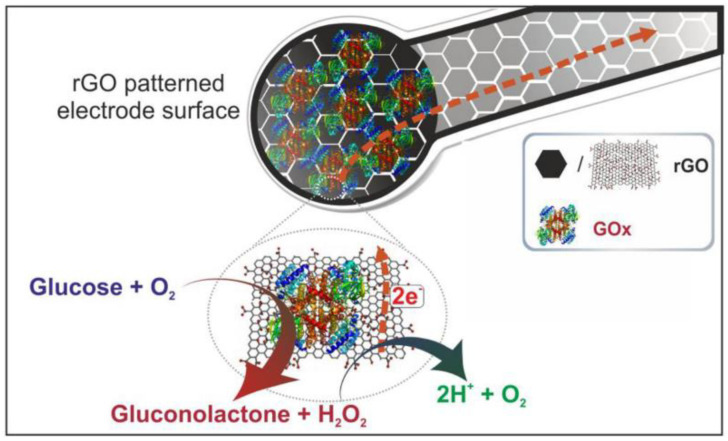
The electrochemical mechanism of glucose oxidation at reduced graphene oxide (rGO)/glucose oxidase is a significant area of study. Reproduced with permission from [136].

An effective biosensor for electrochemical enzymatic determination of glucose can be developed using graphene-based patterns modified with glucose oxidase. The biosensor has a response time of 0.37 s, a sensitivity of 11.2 μA mM^−1^ cm^−2^, and a linear range from 1.0 to 25.0 mM. It also demonstrates a low detection limit of 0.32 mM while maintaining reproducibility, stability, selectivity, and resistance against interference in glucose analysis. The electrochemical mechanism of glucose oxidation at rGO/GOx is depicted in Figure 5 [136]. These results suggest that this platform has the potential to accurately measure glucose levels in human serum and find practical applications in various (bio)sensing systems during sample testing scenarios.

#### 3.4.1. Innovations and Limitations

Recent advancements in biosensor technologies have successfully integrated 2D materials with cellulose, leading to significant improvements in functionality. The ultra-thin nature and extensive surface area of these materials have proven to be pivotal in enhancing the sensitivity of biosensors used for applications such as plasmonic and photoelectrochemical biosensing [137].

By combining nanocellulose with 2D materials, researchers have achieved synergistic enhancements in the performance of biosensors. Nanocellulose offers excellent biocompatibility, a large surface area, and chemical versatility, while 2D materials contribute their unique electrical properties [138]. This combination results in highly sensitive biosensors that can detect even trace amounts of analytes. Furthermore, the mechanical strength provided by nanocellulose ensures the long-term stability and durability of these devices.

Various methods are utilized in material synthesis and processing, each offering distinct advantages and posing specific challenges. For example, Graphene Quantum Dots can be synthesized through top-down and bottom-up approaches, affecting their ultimate properties. Techniques like plasma-enhanced chemical vapor deposition are key in synthesizing MoS_2_ and WS_2_, enabling the creation of consistent, large-area structures. A critical area of research is the integration of bipolar exfoliated reduced graphene oxide with microelectrodes, addressing synthesis and deposition challenges. However, this field faces complexities. The synthesis and processing of these materials, especially when integrating 2D materials with nanocellulose, can influence biosensor performance, impacting sensitivity, specificity, and stability. Advancements in this field necessitate further research to optimize the performance of these sophisticated biosensors. This review consistently emphasizes the primary advantage of these materials: enhanced sensitivity. It’s important to also consider factors like repeatability and the fabrication process, which significantly influence the properties of 2D materials. Despite substantial contributions to the field, uncertainties remain, particularly regarding the relationship between material properties, fabrication methods, and the intricacies within these methods.

#### 3.4.2. Challenges and Future Directions

The challenges of integrating two-dimensional (2D) materials with cellulose for applications such as biosensors are complex and multifaceted. Here are some of the key challenges that need to be addressed:

- Material compatibility and scalability
  - Chemical interaction: The chemical compatibility between 2D materials and cellulose is critical. There may be challenges in achieving a stable chemical interaction that does not compromise the unique properties of the 2D materials or the integrity of the cellulose.
  - Mechanical stability: The integrated structure must be mechanically stable, which is particularly important for flexible applications like electronic skin (E-skin).
  - Large-scale synthesis: Scaling up the synthesis of 2D materials while maintaining their quality and uniformity over large areas is a significant challenge.
  - Integration Techniques: Developing scalable integration techniques that can handle the delicate nature of 2D materials without damaging them is difficult.
- Long-term stability and durability
  - Degradation and sensitivity: Over time and under real-world conditions, there could be degradation of materials or loss of sensitivity, which are crucial for the practical implementation of biosensors.
- Cost and accessibility
  - Synthesis and integration costs: The cost associated with the synthesis and integration of 2D materials and nanocellulose into biosensors might be high, potentially limiting their widespread use.
  - Limited Access: Access to advanced materials and fabrication techniques could be limited, posing a barrier to adoption.
- Biocompatibility and safety
  - Toxicity and immune responses: A comprehensive analysis of biocompatibility and safety is essential, especially for medical applications, to understand potential toxicity or immune responses.
- Performance standardization
  - Inconsistencies in performance: Variations in fabrication methods and materials could lead to inconsistencies in biosensor performance. Standardization protocols are needed for reliable and reproducible results.
- Challenges in integration with AI and IoT
  - Seamless integration: The challenges associated with seamless integration, data processing, and real-time communication between biosensors and AI and IoT technologies need to be addressed [139].

In summary, the integration of 2D materials with cellulose for biosensor applications involves overcoming challenges related to material compatibility, scalability, long-term stability, cost, biocompatibility, performance standardization, and integration with advanced technologies. These challenges must be addressed to ensure the successful development and implementation of these advanced biosensors.

In future research, efforts are focused on developing more sustainable synthesis methods and exploring novel material combinations for broader applications, including screen-printed carbon electrodes modified with graphene quantum dots, MoS_2_, laccase as a caffeic acid biosensor; reduced graphene oxide/molybdenum disulfide/polyaniline nanocomposite-based electrochemical aptasensor for the detection of aflatoxin B1 fabrication [140,141,142].

## 4. Conclusions

This review highlights the recent advancements in biosensor technologies, with a specific focus on integrating two-dimensional materials and nanocellulose. These innovations have resulted in improved sensitivity, specificity, flexibility, and biocompatibility of biosensors. Moreover, the devices created produce a minimal environmental footprint, and they are easy to recycle. The use of these advanced materials has expanded their applications in medical diagnostics, environmental monitoring, and personal health devices.

These developments have transformed the capabilities of biosensors by enabling more precise and early detection of biomarkers. This is particularly important for medical diagnostics and environmental monitoring, where accurate detection is crucially needed. Additionally, the increased flexibility and biocompatibility have paved the way for wearable biosensors that are revolutionizing personal healthcare monitoring.

The potential for advancements in biosensors is immense, and the field is still in its infancy. Ongoing research focuses on improving synthesis techniques, expanding material options, and integrating with artificial intelligence and Internet of Things technologies. These developments will address current challenges and enable innovative applications. The collaboration between material science, bioengineering, and technology plays a vital role in driving the progress of biosensor technologies.

In conclusion, significant advances in material properties have had a profound impact on biosensors. These breakthroughs offer great promise for significant improvements in healthcare and environmental monitoring practices.

## Data Availability

Not applicable.
